# Light chain deposition disease; there are reasons for confusion

**DOI:** 10.12861/jrip.2013.41

**Published:** 2013-10-10

**Authors:** Mohammad-Reza Ardalan

**Affiliations:** ^1^Chronic Kidney Disease Research Center, Tabriz University of Medical Sciences, Tabriz, Iran

**Keywords:** Light chain deposition disease, Plasma cell dyscrasia, Glycosuria

**Implication for health policy/practice/research/medical education:**

Congo red negative nodular deposition of an amorphous, eosinophilic material is the most common pathologic finding in light chain deposition disease in light microscopy. The nodules are a mixture of light chain and mesangial protein and the picture is reminiscent of diabetic nephropathy. Immunoflurescent microscopy usually demonstrates the presence of monotypic kappa light chain. It seems that serum creatinine level greater than 4 mg/dl is a poor prognosis sign for future progress to end stage renal disease in this disease. Therefore, early diagnosis is important.

Light chain deposition disease (LCDD) is characterized by immunoglobulin light chains deposits, most commonly kappa (k) that is neither ﬁbrillar nor Congo red positive. Nodular glomerulosclerosis of LCDD resembles diabetic Kimmelstiel-Wilson. The incidence of LCDD in patients with plasma cell dyscrasia is approximately 5%. Many patients with LCDD are associated with multiple mayeloma (MM), but up to 50% of patients do not have concurrent MM. LCDD frequently have a rapidly progressive renal failure course (1,2).

A 64-year male was referred to our nephrology unit because of elevated serum creatinine level, anemia and glycosuria. Physical examination on admission revealed; Blood pressure; 130/85 mmHg and pulse rate: 88/minute. There was no sign or symptom of heart failure. The initial laboratory examination revealed: WBC: 8900/µl, hemoglobin 8.7 mg/dl, platelet count: 230000/ µl, urine dipstick test revealed glycosuria, hematuria: 2+ and proteinuria: 2+. Serum creatinine: 4.5 mg/dl, serum uric acid; 7.2 mg/dl. Fasting blood glucose: 112 mg/dl but glucose tolerance test was impaired. Twenty four hours urine collection revealed; 2400 mg/dl proteinuria. Serologic study for Hepatitis-B (HBSAg), Hepatitis-C (HCVAb) and HIV infections was negative. Laboratory study for anti-nuclear antibody (ANA), anti-ds DNA antibody and cryoglobulins had negative result. Chest X-ray was normal. Renal ultrasound study disclosed a right kidney size of 120 mm and left kidney size of: 128 mm with increased echogenicity in both sides. The first impression was diabetic nephropathy. Retinal examination was normal. Ultrasound guided renal biopsy was performed. Light microscopic study of the renal biopsy disclosed nodular glomerulosclerosis with prominent amorphous eosinophilic conogo-red negative material deposition. Pathologic picture was compatible with nodular glomerulosclerosis, but there was not any atherosclerotic change or vascular changes compatible with diabetic nephropathy (Figure 1A&B). Immunofluorescence (IF) study of biopsy specimen revealed bright fluorescent staining for kappa light chain. It was also weakly positive for IgG, IgM and C3. Serum electrophoresis revealed a gamma peak. Bone marrow aspiration and biopsy disclosed more than 20% plasma cells infiltration with malignant features. Patients refused to accept any chemotherapy. Two months later, he was admitted in a poor condition and hemodialysis was started urgently. He was non-cooperative to follow dialysis programs or admit any treatment. Three months later, he died during one of his urgent admission while he was in pulmonary edema and sepsis.

**Figure 1 (A & B):**
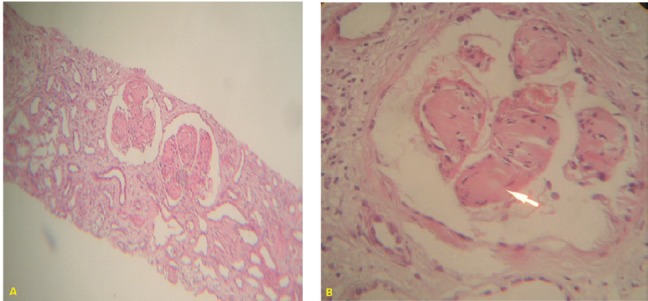
Renal biopsy specimen shows global and amorphous Nodular glomerulosclerosis of Kappa Light chain deposition, resembling diabetic Kimmelstiel-Wilson.

In this patient detection of glucosuria, impaired glucose tolerance test and bilateral enlarged kidneys were compatible with diabetic nephropathy. Light microscopic findings of nodular lesion were also in favor of diabetic nephropathy. Immune fluorescent findings of kappa light chain deposit within the nodules and gamma spike in serum electrophoresis confirmed the diagnosis of LCDD. Renal manifestation of plasma cell dyscrasia includes; cast nephropathy (myeloma kidney), amyloidosis, monoclonal immunoglobulin deposition diseases that includes both LCDD and heavy chain deposit disease(HCDD), light chain fanconi syndrome, cryoglobulinemic glomerulonephritis and Waldenstrom macroglobulinemia with intracapillary IgM deposition. Immunotactoid glomerulopathy which commonly associated with lymphoproliferative diseases resembles Membranous nephropathy in light microscopy and it is a congo-red negative organized glomerular deposits (3). Acute interstitial nephritis could be the heralding sign of multiple myeloma (4). Congo red negative nodular deposition of an amorphous, eosinophilic material is the most common pathologic finding in LCDD in light microscopy. The nodules are a mixture of light chain and mesangial protein and the picture is reminiscent of diabetic nephropathy. Immunoflurescent microscopy usually demonstrates the presence of monotypic kappa light chain. Heavy chain deposit disease (HCDD) could also manifest as nodular lesion, however IF study differentiate these two entities (5) Light chain is absorbed by mesangial cells and decreases the secretion of matrix methaloproteinase-7( MM-7), but conversely increases the secretion of tenascin by this cells. The first effect is not seen by light chains of AL-Amyloidosis or in cast nephropathy (3). The typical clinical picture of LCDD is similar to rapidly progressive glomerulonephritis. Proteinuria is a combination of light chain and albumin. Light chain proximal tubular involvement and fanconi syndrome could manifest with glycosuria, aminoaciduria, phosphaturia, and hyperuricosuria, that is a consequence of direct tubular toxicity and intra-lysosomal crystalline deposits of light chains (3-5). The treatment of LCDD is difficult and patient appears to benefit from the same chemotherapy as that given for multiple myeloma.

It seems that serum creatinine level greater than 4 mg/dl is a poor prognosis sign for future progress to end stage renal disease. Therefore, early diagnosis is important.

## Author’s Contribution

MRA is the single author of this paper.

## Conflict of interests

None to declare.

## Ethical considerations

Ethical issues (including plagiarism, data fabrication, double publication) have been completely observed by the author.

## Fund/Support

No financial support by any institution.
